# A Review of Clinicopathological Characteristics and Treatment of Solid Pseudopapillary Tumor of the Pancreas with 2450 Cases in Chinese Population

**DOI:** 10.1155/2020/2829647

**Published:** 2020-07-01

**Authors:** Jihang Yao, He Song

**Affiliations:** ^1^Department of Gynecology of the First Hospital of China Medical University, Nanjing Street 155 Shenyang 110001, China; ^2^Department of Gastrointestinal Surgery of the First Hospital of China Medical University, Nanjing Street 155 Shenyang 110001, China

## Abstract

**Background:**

Solid pseudopapillary tumor of the pancreas (SPTP) has been reported as a rare disease with low malignant potential. The aim of this study was to summarize experiences of the diagnosis and treatment for the patients reported in the Chinese population.

**Method:**

2450 SPTP cases reported in English and Chinese literature before Jan 2020 were for our review and analysis retrospectively.

**Result:**

There are 389 male cases and 2061 female cases, and the ratio of male/female was 1 : 5.3. The average age was 29.3 years. The main clinical symptoms were upper abdominal pain and bloating discomfort in 51.6% of the cases and epigastric mass. 38.6% of the tumor was located at the head of the pancreas and 55.4% at the body and tail of the pancreas. The most frequent operative styles were tumor enucleation (38.4%). Pathology showed that the average diameter of the tumor was 8.2 cm and 12.3% of SPTP was malignant. 98.3% of cases had favorable survival.

**Conclusions:**

SPTP is a rare indolent tumor occurring mainly in young women, and the main clinical performances are abdominal mass and abdominal pain; most tumors are distributed at the head and the tail of the pancreas; the prognosis after complete resection is excellent.

## 1. Background

SPTP is a low malignant potential tumor of the low incidence. Recently, the reports of SPTP have increased markedly since 2000. Before 2010, most articles focused on the case reports or case series involving fewer than 20 patients. After 2010, most articles concentrated on the observational study of large-scale case series of more than 50 patients. Papavramids had summed up 718 SPTP patients in English literature, mainly from USA, Europe, and Japan [1]. However, studies have shown SPTP occurred more frequently in eastern countries [2–5]. A total of 390 cases [6] and 553 cases [7] were reviewed in the SPTP reported in Chinese literature. A nationwide survey from China [8] and South Korea [9] revealed that SPTP ranked as the first (31.7%) and the third (18.3%) common in the pancreatic cystic tumors (18.3%). Despite the increasing knowledge of the disease, biological features, pathogenesis, and effective therapeutic strategies remain unclear. Due to the Chinese population of a large sample, the aim of this study is to execute the systematic review of all the available researches of SPTP published in the English and Chinese literature for summarizing clinicopathological and therapeutic experiences in the Chinese population.

## 2. Methods

### 2.1. Document Collection

SPTP-related literatures covered in PubMed, Web of Science, SinoMed database, Wanfang Data Corp., and China National Knowledge Infrastructure (CNKI) before Jan. 2020 were retrieved. Indexing terms used for retrieval were for solid pseudopapillary tumor of the pancreas, solid pseudopapillary neoplasm, cystic solid tumors, solid epithelial tumors, papillary cystic tumor, cystic papillary epithelial neoplasm, and Frantz tumor, China mainland[affiliation], Taiwan[affiliation], Hongkong[affiliation], and Macau[affiliation]. For the clinical research papers, by means of inspection carefully and removal of the irrelevant or duplicate literature and foreign abstract translations, we got 378 original papers, including 201 case reports, 67 imaging diagnosis, 103 clinical pathological analysis, 26 literature reviews, and 40 other papers (immunohistochemical study or mechanism, etc.). The first related report of 3 cases was investigated in 1984 [10]. The largest single-study case series comprised of 243 cases [11].

### 2.2. Criteria of Case Screening

**Figure 1:**
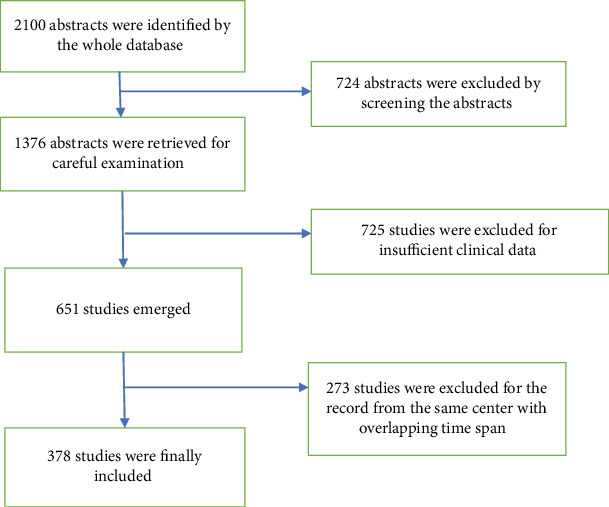
Flow chart of article processing-related SPTP.

Publications of any study design with a definite histologic diagnosis of SPTP and a description of patient clinical characteristics (age and/or gender and/or symptoms and/or localization and/or size and/or metastasis and/or treatment and/or pathology and/or follow-up) were included. Each case representing six of the above 9 items was defined as well-documented. Besides the English literature, the literature in Chinese from MEDLINE and from the regional high-volume medical center was selected. To avoid duplication of cases, the demographic and clinical characteristics of patients were cross-referenced originally by the hospital or center from which the cases arise. If multiple case series were published from the same hospital or center with the overlapping study periods, the English publications with the latest and largest number of cases were used. If a duplicate case was reported in separate case reports, the case report with the most relevant clinical data was used. Studies reported as abstracts were excluded due to insufficient information. The corresponding authors were sought for additional specific information via e-mail. At last, we got 235 reports and 2450 clinical case information for analysis. The flow chart is listed in Figure 1. A great number of studies did not include each item in the data extracted. The frequency of findings was based on the number of patients in whom the finding was frequency related to each variable listed in Table 1.

## 3. Results

### 3.1. Common Information

A total of 2450 cases included 389 male cases and 2061 female cases, and the ratio of male/female was 1 : 5.3. There was a clear age record in 2320 cases; meanwhile, the youngest is 3-year-old and the eldest is 82-year-old, and the average age was 29.3 years.

### 3.2. Clinical Manifestations

Within 2450 cases, there were 2385 well-documented records for the patients' symptoms. The clinical presentation was upper abdominal pain or bloating discomfort in 1230 cases (51.6%), abdominal mass in 960 cases (40.2%), incidentally detection or asymptomatic in 902 cases (38.6%), nausea and vomiting in 304 cases (12.7%), diarrhea in 167 cases (7.0%), obstructive jaundice in 72 cases (3.0%), and abdominal trauma in 45 cases (1.9%). A small number of patients were with acute pancreatitis, upper gastrointestinal bleeding, obvious loss of weight, intestinal obstruction, etc. The symptoms were nonspecific, and the coexistence of two or more symptoms was often found.

### 3.3. Imaging and Laboratory Tests

Within 2450 cases, there were 2208 well-documented for patients' imaging examination. The three most common forms of abdominal imaging were CT, ultrasound, and MRI and accounted for 2021 cases (92.5%, 2021/2208), 1370 cases (62.0%, 1370/2208), and 970 cases (43.9%, 970/2208). Calcification in the tumor was reported for 716 patients, which accounted for 32.4% (716/2208). Positron emission tomography/computed tomography PET/CT, endoscopic ultrasound (EUS), endoscopic retrograde cholangiopancreatography (ERCP), digital subtraction angiography (DSA), and fine-needle aspiration cytology (FNAC) were performed in 82 cases, 38 cases, 31 cases, 18 cases, and 40 cases. Among the 50 patients performed by FNAC, 40 cases were diagnosed as SPTP, 4 cases were diagnosed as dubious tumor tissue, and another 6 cases were misdiagnosed as islet cell tumor.

There were 603 well-documented records including liver function, blood, serum amylase, and tumor markers. The level of tumor markers, including AFP, CEA, CA199, CA125, CA242, CA50, CA724, and CA153, was slightly increased in 62 patients, but only 22 cases were diagnosed as malignant SPTP by pathology.

### 3.4. Treatment

There were 2387 well-documented records of patients' tumor location. Within 2387 cases, 924 cases (38.6%) were distributed on the pancreatic head, 1324 cases (55.4%) were distributed on the pancreatic body and tail, 120 cases (5.0%) were distributed on the pancreatic neck, and 24 cases (1.0%) were outside the pancreas. Nearly all of them were solitary. Interestingly, there were 5 multicentric cases. The choice of surgical procedures depended on the position of the tumor in the pancreas. Pancreatic resections were reported in 2110 patients. There were 812 patients (38.4%) who underwent enucleation, 428 patients (20.2%) who underwent pancreatoduodenectomy, 112 patients (5.3%) who underwent duodenum-preserving partial pancreatic head resection, 382 patients (18.1%) who underwent distal pancreatectomy and splenectomy, meanwhile 5 cases combined with left nephrectomy, 311 patients (14.7%) who underwent distal pancreatectomy with preserving spleen, 40 cases (1.9%) who underwent central pancreatectomy, 9 cases (0.4%) who underwent total pancreatectomy, 4 cases (0.2%) who underwent cytoreductive surgery, and 12 cases (0.6%) who underwent a palliative operation. The infiltrated portal vein/superior mesenteric vein was reconstructed with a vein graft after pancreatoduodenectomy in 15 patients. Synchronous metastasectomy of the liver, gastrectomy, and colectomy were performed, respectively, in the 12, 5, and 6 patients with liver metastasis, gastric involvement, and colonic involvement. The surgery was performed via the open procedure in 1935 cases, whereas 103 cases and 72 cases were, respectively, performed in laparoscopic and in the robotic approach. 14 cases had a pathological biopsy, 5 cases had transcatheter arterial chemoembolization (TACE), 9 cases had radiotherapy or chemotherapy treatment (meanwhile, 4 cases had palliative operation noted above), 2 cases received hyperthermic intraperitoneal chemotherapy (HIPEC), 2 cases received the implantation of a radioiodine-125 particle, and 1 case received interventional arterial embolization.

### 3.5. Pathological and Immunohistochemical Characteristics

Within 2450 cases, tumor size was recorded in 2093 patients. The average diameter was 8.2 cm ± 1.4 (0.2-30 cm). Tumors were grossly large, spherical, well-demarcated, and well encapsulated. There were 1520 cases with description for tumor capsule, meanwhile the complete capsule for 1012 cases (66.6%), the incomplete capsule for 315 cases (20.7%), and the noncapsule for 193 cases (12.7%). Microscopically, tumor cells arranged around fibrovascular stalk forming a pseudopapillary pattern, focal areas of hemorrhage, and necrosis could usually be found. Immunohistochemical staining showed that the positive rate of *α*-ACT, Vinmentin, *α*-AT, NSE, progesterone receptor (PR), Synaptophysin, CD56, CD10, CD99, androgen receptor (AR), TFE3, P504s, claudin-5, CgA, ER, and E-cadherin was 96.5% (1856/1923), 96.9% (1834/1892), 96.7% (1812/1874), 91.1% (1802/1978), 94.5% (1605/1698), 90.1% (1408/1562), 94.1% (94/423), 79.9% (467/571), 82.6% (456/552), 13.3% (4/30), 94.7% (71/75), 100% (67/67), 100% (37/37), 15.3% (56/365), 11.7% (17/145), and 0% (0/87), respectively. Immunohistochemical staining of Ki-67 was detected in 412 patients. The Ki-67 index ranged from 0 to 30%. 2450 cases were pathologically confirmed as SPTP; 302 cases (12.3%) were diagnosed as malignant as follows: vascular infiltration was identified in 104 cases, lymph node involvement in 12 cases, pancreatic parenchyma and fat invasion in 88 cases, perineural invasion in 29 cases, adjacent organ invasion in 16 cases, distant metastasis in 43 cases, and nuclear atypia or a high mitotic rate in 10 cases.

### 3.6. Postoperative Complications and Follow-Up Results

1788 cases were well recorded in postoperative complications as follows: pancreatic fistula in 294 cases, pancreatitis in 14 cases, alimentary tract hemorrhage in 8 cases, pseudocyst in 2 cases, chylous fistula in 8 cases, bile leakage in 10 cases, gastroparesis in 121 cases, and diabetes in 20 cases.

In 1834 cases of the total with follow-up information available with the follow-up range time (2-240 months), 98.3% (1802/1834) of the cases had good survival. 32 cases (1.7%, 32/1834) died as follows: 25 cases died from SPTP, 5 cases died from other diseases, and 2 cases died from an unclear reason. Recurrence was reported in 65 patients (3.5%), with a mean time to recurrence of 23.6 months. Twenty of them were operated for the second or third time and achieved good survival.

## 4. Discussion

In 1959, Frantz first reported the disease and called it “benign or malignant papillary tumor of the pancreas.” According to their morphological characteristics, it used many descriptive names, respectively, later, such as papillary epithelial neoplasm, cystic and solid tumors, solid epithelial tumors, cystic papillary epithelial neoplasm, and Frantz tumor. These different names reflect the morphological diversity of tumor tissue at the same time. In 1996, WHO defined SPTP as the borderline tumor of the pancreas or uncertain malignant potential tumor and uniformly named as the solid pseudopapillary tumor. Papavramidis and Papavramidis [1] and Law et al. [12] retrospectively reported 718 cases and 2744 cases of SPTP in the English publication worldwide. Kuo et al. [10] reported this tumor in 1984 for the first time in China. Yu et al. [13] and Pancreatic Surgery of Chinese Academic Society of Young [8], respectively, analyzed 553 cases and 713 cases in the Chinese population. These two reviews were not incorporated into the current analysis for lack of specific clinical details, but a hand search of the related references was performed, and the original studies in the references were included. In recent years, as the constant awareness of this disease, related literature in both English and Chinese has been continuously increasing, as the SPTP were likely to occur in the eastern country. We collected 2450 Chinese cases before Jan 2020. To our knowledge, the current review represents the largest number of patients with SPTP reported in the English and Chinese publications. We hoped to further summarize its clinical data and guide the clinical practice by means of increasing cases; meanwhile, it would be helpful for the incidence of the SPTP in the epidemiological analysis in the Chinese population.

SPTP is a rare exocrine tumor of the pancreas and takes up 1%-2% of all pancreatic tumors. Papavramidis and Papavramidis [1] indicated that a ratio of male/female in 690 patients was 1 : 9.78 and the average age was 21.9 years, while Law et al. [12] showed that the ratio of male to female in the patients of 2744 cases was 1 : 7.3 and the average age was 28.5 years. In our statistics of 2450 cases, the disease often occurred frequently in Chinese young women with the average year of 29.3, and the male/female ratio of 1 : 5.3, suggesting that gender ratio and the age of Chinese patients were slightly different compared to the world population. A majority of cases were reported in the Chinese high-volume medical centers; the SPTP was mainly distributed in the coastal and southern regions of China which own high-quality medical and economic standards (shown in Table 2).

The presentation of SPTP is usually nonspecific. Similar to Western literature, our data showed abdominal discomfort or pain found in 51.6% of the patients and abdominal mass was seen in 40.2% of the patients. Some patients (38.6%) are asymptomatic, and tumor was incidentally detected by imaging examination. A retrospective analysis of 109 patients showed the average tumor size of the asymptomatic SPTP group was significantly smaller than that of the symptomatic SPTP group [14]. For the most tumor size which is big with the average diameter of 8.2 cm, some patients presented symptoms of tumor compression affecting the alimentary tract, such as nausea and vomiting. In some severe cases, patients could be manifested as intestinal obstruction and pancreatitis. For insufficient blood supply of tumor, some cases were manifested as anemia due to internal bleeding from ischemic necrosis of tumor, or even in the severe situation manifested as a shock due to spontaneous rupture of the tumor. It is necessary to note that even if the tumor was located at the head of the pancreas, it rarely caused obstructive jaundice. Although the 36% tumor [12] and 38.6% tumor in our data were distributed at the head of the pancreas, only 10% (10/97) cases and 3.0% cases represented obstructive jaundice, which may be related to the tumor ectogenous growth way.

Imaging examination is important for tumor detection and location. CT is the most predominant imaging procedure, which is better than ultrasound in the accuracy of detection. In addition, the tumor calcification by ultrasonography and CT was found in nearly one-third of the patients, suggesting this specific image may be beneficial for diagnosis. CT typically showed a large well-circumscribed, heterogeneous mass with a wide range of appearance from solid to cystic components, demarcated by a capsule with occasional hemorrhage or/and calcification [15]. SPTP in male patients is often displayed as solid and nearly solid [16]. Some studies [16–18] showed the average tumor size in males was significantly smaller than that in females. MRI is superior to CT in differentiating the internal structure of tumor, such as hemorrhage, cystic degeneration, and tumor capsule. Solid tumors and solid parts of mixed tumors were T2 hyperintense and T1 hypointense and represented progressive enhancement [16, 17]. In recent years, texture analysis based on the radiomics is very popular for differentiation among the pancreatic tumors. MRI texture analysis can sensitively distinguish between nonfunctional pancreatic neuroendocrine neoplasms and SPTP [19]. All the 82 SPTP cases were performed by PET/CT, whose SUVmax values were mainly more than 2.5 with the range of 2.4-44.8. Their accumulation of fluorine-18 fluorodeoxyglucose (FDG) displayed positive. FDG uptake of SPTP may be related to tumor cellularity, proliferative index, or histological malignancy [20]. SPTP could be differentiated from pancreatic ductal adenocarcinomas and neuroendocrine tumors by the lower SUVmax [21]. DSA usually represented a hypovascular tumor but is not helpful for the diagnosis. Endoscopic ultrasound usually showed a hypoechoic and heterogeneous mass. A combination endoscopic ultrasound with needle aspiration biopsy cytology (FNAC) could enhance the preoperative diagnostic yield. Furthermore, EUS-FNA is more effective than CT in differentiating malignant pancreatic mass. Our study showed the diagnostic accuracy performed by FNAC was 75% (30/40) consistent with the accuracy of 69.5% [12] and no complications occurred such as tumor disseminating and pancreatic leakage. In laboratory tests, parameters were commonly within normal scope, so the routing laboratory parameters and tumor markers are of no value.

In our study, the predominant localization of tumor is the body and tail of the pancreas, followed by the head and the neck, which is consistent with the previous report of [1, 12, 13]. SPTP is usually solitary and single-focal mass; however, 3 cases of multifocal SPTP had similar clinicopathological characteristics [22]. Deserved to be mentioned, there were 24 cases in the extrapancreatic sites as follows: mesocolon (2 cases), retroperitoneum (11 cases), omentum (5 cases), duodenum (3 cases), and ovary (3 cases). These extrapancreatic SPTP substantiated by the migration of the pancreas during embryogenesis [23] are likely to represent a favorable clinical course similar to the normal SPTP.

The preferred treatment of SPTP is surgery. The selection of the surgical approach depends on the intraoperative pathological frozen section, tumor location, and infiltration of surrounding structures. For the tumor's ectogenous growth tumor, enucleation is the first choice. Jin et al. [24] regarded indication for the enucleation is the SPTP which was located on the anterior or posterior of the pancreatic peripheral surface and far (>3 mm) from the common bile duct and main pancreatic duct. Enucleation of SPTP displayed to be more feasible and safe for exocrine function with no increased risk of tumor recurrence compared to conventional pancreatic resection [25]. More aggressive surgical determination should be adopted when PV/SMV or adjacent organ involvement or hepatic metastasis is indicated on preoperative imaging [26, 27]. Most of advanced and metastatic SPTP represented a favorable survival. In addition, 24 cases of SPTP were found outside the pancreas, so careful exploration is particularly essential in the operation. Very few of SPTP emerged lymph node metastasis, so extensive lymphatic dissection is not necessary [1, 12, 28]. Tang et al. reported 4 (8.2%) patients represented nodal metastasis among 49 patients involved in lymphadenectomy, while peripancreatic lymphadenopathy was associated with malignancy in SPTP patients [29]. Within 302 cases of malignant tumor of this paper, lymph node metastasis was found in only 12 cases. Western literature suggested that for the patients returning to hospital due to tumor recurrence or metastasis of the liver or intraperitoneal, etc., it was suggested not only to conduct the radical resection or cytoreductive surgery but also to use hyperthermic intraperitoneal chemotherapy (HIPEC) [23]. For the unresectable or recurred cases, radiofrequency ablation (RFA) [30], gamma-knife treatment, transcatheter arterial embolization [31], radiotherapy, and chemotherapy should be considered [32]. Adjuvant therapy is successful in 3 cases. Notably, chemotherapy regimen with floxuridine and oxaliplatin or etoposide and cisplatin or S-1 might be the appropriate choice for chemotherapy [13, 31, 32]. However, the small number of cases is required to add more medical evidence to draw the conclusion for the role of adjuvant therapy and embolization for SPTP. In recent years, as laparoscopic and robotic technology has been developing, due to minimally invasive advantages, it was expected to become one of the ways of SPTP treatment because tumors were generally benign and represented thick fibrous capsule. The clinical outcome of laparoscopic and robotic excision of pancreatic tumors in the Chinese high-volume medical center was satisfactory [24, 33, 34]. Furthermore, the robotic technique seemed to be more efficient and secure than a laparoscope among properly selected patients [35].

Diagnosis of SPTP mainly depends on the pathology and immunohistochemistry.

Pathological diagnosis is mainly based on its cystic structure and characteristic pseudopapillary structure. Part of the tumor violating blood vessels and normal pancreas, emergence of giant cell tumor, increased mitotic, diameter of tumor bigger than 5 cm, and obvious necrosis are diagnosed as malignant [36]. However, the final diagnosis is based on microscopic appearance. As a previous description [37], the microscopic appearance of SPTP in the majority of the papers showed mainly that the solid areas were composed of monotonous polygonal epithelioid cells. Some areas showed more extensive stromal fibrosis, with round aggregates of perivascular hyalinized stroma imparting a cylindromatous appearance. In the pseudopapillary regions, the cells located away from the small vessels appeared to have dropped away, leaving an irregular cuff of cells surrounding each vascular core. Clusters of cells demonstrated large eosinophilic cytoplasmic globules. The nuclei were generally uniform and round to oval. The areas between the pseudopapillary structures were filled with red blood cells. In addition, our study showed the positive expression rate of *α*-ACT, Vinmentin, *α*-AT, NSE, progesterone receptor (PR), Synaptophysin, and CD56 was higher in immunohistochemistry and widely used, especially the positive expression rates over 90%, which had some reference value for the diagnosis. On the other hand, some proteins were detected negative for expression. Yu et al. showed that E-cadherin was a loss of expression in all of 37 cases [38]. Moreover, there were some biomarkers related to the gender hormone in a distinct expression. Wang et al. reported that negative PR expression was significantly associated with poorer disease-free survival (DFS) and disease-specific survival (DSS) [39]. Zou et al. reported that nearly half of the male cases showed positive immunoreactivity for androgen receptor (AR), which is significantly different from female cases [40]. Several gene expression is valuable for differentiation of SPTP and other pancreatic tumors. Jiang et al. indicated that 71 (94.67% of 75) cases of SPTP showed positive expression for TFE3, which could be easily differentiated from the pancreatic neuroendocrine tumor (PNET) and pancreatic ductal adenocarcinoma [41]. Zheng et al. regarded immunostaining positive claudin-5 for 37 cases of SPTP and negative for 21 cases of PNET could differentiate between these entities [42]. Chen et al. and Shen et al., respectively, analyzed 43 and 26 cases of SPTP showing granular cytoplasmic expression of P504s, whereas all the cases of PNET and pancreatic acinar cell carcinoma (PACC) were negative. P504s is an effective marker for SPTP with distinction from PNET and PACC [43, 44]. Most Chinese scholars defined the malignancy of SPTP by the WHO criteria such as angioinvasion, perineural invasion, or deep infiltration into the surrounding tissue or metastasis. The tumor cells may invade the capsule and metastasize, most commonly to the liver [27]. Attributable to the debatable criteria of SPTP malignancy, a few risk factors of predicting malignancy were controversial. Xu et al. found that [45] distal location of SPTP indicated malignancy, whereas other studies [13, 46, 47] did not show any relation between location and malignancy. Ye et al. [48] regarded that SPTP with incomplete capsule had larger tumor size compared to the complete capsule group. Song et al. [46] revealed that an incomplete capsule was not only the predictive factor of malignancy but also the significant predictor of disease-specific survival, while Wang et al. indicated that lack of complete capsule and age > 40.5 years were independent risk factors of malignant SPTP [49]. A meta-analysis [50] involving the English articles revealed that malignant SPTP tended to be larger in diameter and younger in age than benign type. Some researchers considered Ki-67 could be recommended for another immunohistochemical parameter for the diagnosis of malignancy [51]. Two cases of 13 pathological confirmed malignant SPTP is detected to be more than 4% for Ki-67, which was significantly associated with recurrence [47]; on the other hand, low proliferative index or negative Ki-67 staining result had relatively lower FDG uptake in the PET-CT scan [20]. Grossly, there is no consensus on the standard parameter to predict the malignancy of SPTP.

China's largest cross-sectional study showed the incidence of postoperative complications of pancreatic cystic tumor with a total of 2251 cases is 46.0% (935/2261). Among the 2251 cases of pancreatic cystic tumor, the incidence of postoperative complications for serous cystic neoplasm (SCN), mucinous cystic neoplasm (MCN), SPTP, and intraductal papillary mucinous neoplasm (IPMN) was 50.3%, 44.1%, 43.8%, and 43.7%, respectively [8]. However, the incidence in our study was 26.7% (477/1788). There was a little discrepancy between the two data, and it probably should be the included cases in our study which failed to report the detailed clinical outcome. Some scholars indicated that enucleation might be an effective approach to reduce postoperative complications [25].

Overall, most prognoses with SPTP were favorable and less than 5% of patients had local recurrence and metastasis, which showed an agreement with the previous study [1, 12, 13]. The largest sample study from the single center showed 4 cases out of 243 cases died due to recurrence, with a 5-year survival rate of 98.4% [11]. High NLR were not only associated with worse recurrence-free survival (RFS) but also the only independent predictor of malignant SPTP [52]. Negative progesterone receptor (PR) result was significantly associated with poorer disease-free survival (DFS) and disease-specific survival (DSS) [39]. The multicenter of large-scale clinical data is still requested to explore the risk factor affecting survival.

SPTP is involved in the Wnt/*β*-catenin, Hedgehog, Notch, and androgen receptor signal pathways. Genetically, SPTP have distinct diver gene in comparison to pancreatic ductal adenocarcinoma. Guo et al. [53] conducted whole-exome sequencing of paired SPTP tissues from nine patients and found CTNNB1 playing the key part in the network of cancer promotion, which might interact with USP9X, EP400, HTT, MED12, and PKD1. Generally, we regarded the SPTP is not hereditary. It is striking to note that there was a report [54] on the familiar aggregation that three SPTP patients of one family were detected to present Leu104Val mutation of protease serine 1.

The main limitation of this study is that the quality of some reports is poor because most publications are case reports without prospective analysis or standard randomized study. Some reports did not include all the outcomes of the patients. However, much attention was paid to avoid duplication of cases. So there would be divergence about standard clinicopathological data. Further study should be dependent on detailed clinical information. Despite the limitation, this is the largest review of SPTP in the Chinese population, meanwhile a retrospective review of the largest number of cases in the eastern countries.

## 5. Conclusion

We have analyzed 2450 SPTP cases in the Chinese population over 36 years in this study. In brief, SPTP is a low-grade malignant potential tumor. Clinical manifestations had no specificity, imaging examinations are contributed to tumor location, and diagnosis relies on pathology. Surgery is the mainstay of treatment. For the patient of recurrence or metastasis, aggressive surgery and comprehensive treatment are entitled to receive a satisfactory prognosis.

## Figures and Tables

**Table 1:** Patient demographics and tumor characteristics.

Variable	Number (*n*)	Frequency (%)
Gender	2450	
Male	389	15.9%
Female	2061	84.1%
Age (year)	2320	
Mean + SD	27.1 ± 12.3	
Symptom	2385	
Abdominal pain	1230	51.6%
Abdominal mass	960	40.2%
Incidentaly detection	902	38.6%
Nausea	304	12.7%
Diarrhea	167	7.0%
Obstructive jaundice	72	3.0%
Abdominal trauma	45	1.9%
Usage rate of imaging	2208	
CT	2021	92.5%
Ultrasonograph	1370	62.0%
MRI	970	43.9%
Tumor location	2387	
Head	924	38.6%
Body and tail	1324	55.4%
Neck	120	5.0%
Extrapancreatic lesion	24	1.0%
Surgery	2110	
Enucleation	812	38.4%
Pancreatoduodenectomy	428	20.2%
Duodenum-preserving		
Pancreatic head resection	112	5.3%
Distal pancreatectomy and splenectomy	402	19.0%
Distal pancreatectomy with preserving spleen	331	15.9%
Central pancreatectomy	40	1.9%
Total pancreatectomy	9	0.4%
Cytoreductive surgery	4	0.2%
Palliative surgery	12	0.6%
Operation style	2110	
Open surgery	1935	91.7%
Laparoscopy	103	4.8%
Robotic surgery	72	3.4%
Tumor size (cm)		
Mean + SD	8.2 ± 1.4	
Tumor capsule	1520	
Complete capsule	1012	66.6%
Incomplete capsule	315	20.7%
Noncapsule	193	12.7%
Positive rates		
*α*-ACT	1856	96.5% (1856/1923)
Vinmentin	1834	96.9% (1834/1892)
*α*-AT	1812	96.7% (1812/1874)
NSE	1802	91.1% (1802/1978)
PR	1605	94.5% (1605/1698)
Synaptophysin	1408	90.1% (1408/1562)
CD56	398	94.1% (408/423)
CD10	467	79.9% (467/571)
CD99	456	82.6% (456/552)
AR	4	13.3% (4/30)
TFE3	71	94.7% (71/75)
P504s	67	100% (67/67)
Claudin-5	37	100% (37/37)
CgA	56	15.3% (56/365)
ER	17	11.7% (17/145)
E-cadherin	0	0%(0/87)
Pathological type	2450	
Benign	2148	87.7%
Malignant	302	12.3%
Vascular infiltration	104	
Lymph node involvement	12	
Pancreatic parenchyma and fat invasion	88	
Perineural invasion	29	
Adjacent organ invasion	16	
Distant metastasis	43	
Nuclear atypia	10	

**Table 2:** Reported case series of large-scale (>50) cases of SPTP in the English and Chinese literature.

Publication year	Author	Institution	Cases	Male	Female	Age	Median follow-up (month)
2020	Lin et al. [55]	Fujian Medical University Union Hospital	60	12	48	32.2	47 (3-118)
2019	Wang et al. [49]	The Affiliated Hospital of Nanjing University of Chinese Medicine	122	26	96	33.5	NA
2019	Yang et al. [52]	Huashan Hospital	113	23	90	35	49 (2-167)
2019	Liu et al. [11]	Fudan University Shanghai Cancer Center	243	62	181	35.3	46 (0-118)
2019	Wu et al. [56]	Peking University Cancer Hospital	54	9	45	32.6	60 (4-120)
2019	Hu et al. [14]	Affiliated Hospital, Jiangnan University	109	19	90	30	62 (4-97)
2019	Zhan et al. [57]	Qilu Hospital, Shandong University	91	13	78	28.8	(2-121)
2018	Wang et al. [39]	Wuhan Union Hospital	76	17	59	30	23.5
2018	Jiang et al. [41]	First Affiliated Hospital of Xi'an Jiaotong University	75	10	65	30.9	NA
2018	Wang et al. [58]	Pancreas Center of Nanjing Medical University	97	12	85	31.6	54
2017	Xu et al. [45]	Zhongshan Hospital, Fudan University	121	28	93	33.7	NA
2017	Yang et al. [31]	Henan Tumor Hospital	55	7	48	33	53
2017	Song et al. [46]	The First Hospital of China Medical University	53	7	46	35.4	48
2016	Xu et al. [59]	General Hospital of PLA	148	31	117	NA	32.6
2015	Zhang et al. [28]	Shandong Provincial Hospital	62	6	56	26	46
2015	Yu et al. [13]	Zhejiang Cancer Hospital	97	4	93	31.2	70.1
2014	Wang et al. [27]	Peking Union Medical College Hospital	187	28	159	30	30
2014	Cai et al. [60]	West China Hospital	116	16	100	Female 33.1; male 43.1	58
2014	Hu et al. [18]	The Affiliated Renmin Hospital, Jiangsu University	102	16	86	Female 28.7; male 38.5	NA
2013	Chengqian et al. [61]	Cancer Institute and Hospital, Chinese Academy of Medical Sciences	53	5	48	32.7	NA
